# Molecular Docking for pharmacokinetics and drug‐likeness of Naringenin and its protective effects against streptozotocin induced cognitive deficits in mice

**DOI:** 10.1002/alz.086392

**Published:** 2025-01-03

**Authors:** Monu Yadav

**Affiliations:** ^1^ Amity Institute of Pharmacy, Amity University, Gurugram, Haryana India

## Abstract

**Background:**

The current study aimed to investigate the chemical interaction of naringenin with the possible receptors and enzymes involved in the pathogenesis of cognitive deficits and tested their ADME and toxicity. Furthermore, *in‐vivo* studies have also done to evaluate the effect of naringenin and its nanoparticles on STZ‐induced cognitive decline in mice.

**Method:**

Naringenin were evaluated against the active sites of β‐secretase 1 (PDB: 3UQU), human insulin‐degrading enzyme (PDB: 4RE9), insulin receptor tyrosine kinase (PDB: 1IR3), glycogen synthase kinase‐3 β (PDB: 3L1S), phosphoprotein phosphatase 2A (PDB: 3P71), human superoxide dismutase I (PDB: 5YT0), catalase‐3 (PDB:3EJ6), and human acetylcholinesterase (PDB: 4EY7) in comparison of rivastigmine using molecular docking studies. Further, the naringenin and rivastigmine were subjected to test their pharmacokinetic and lead‐likeness properties by employing the Swiss ADME web server. Moreover, a toxicity study has been performed for both naringenin and rivastigmine with the help of a Protox‐II web server.

**Result:**

Naringenin has a better binding affinity than rivastigmine with all the potential receptors. In *in‐vivo* behavioral, biochemical mitochondrial and histopathological studies, naringenin nanoformulation showed more significant cognitive enhancement results as compared to pure naringenin when studied against STZ induced cognitive deficits.

**Conclusion:**

The results demonstrated that it would be worthwhile to explore more naringenin nanoformulation to expose the therapeutic effects for the treatment of cognitive deficits and neuroprotecive effects.

